# Risk of Congenital Heart Disease in Newborns with Prenatal Exposure to Anti-depressant Medications

**DOI:** 10.7759/cureus.4673

**Published:** 2019-05-15

**Authors:** Pooja H Desai, Priyank J Yagnik, Nancy Ross Ascuitto, Parna Prajapati, Steffan Sernich

**Affiliations:** 1 Pediatric Cardiology, University of Kansas School of Medicine, Wichita, USA; 2 Pediatrics, University of Kansas School of Medicine, Wichita, USA; 3 Pediatric Cardiology, Ascuitto Pediatric and Adult Congenital Cardiology, New Orleans, USA; 4 Child & Adolescent Psychiatry, Virginia Commonwealth University, Richmond, USA; 5 Pediatric Cardiology, Louisiana State University Health Science Center, New Orleans, USA

**Keywords:** antidepressant, congenital heart disease, ssri, fetal echocardiogram, fluoxetine, citalopram, ventricular septal defect, venlafaxine

## Abstract

Introduction

It is uncertain whether the use of selective serotonin-reuptake inhibitors (SSRI) and other anti-depressants during pregnancy is associated with an increased risk of congenital heart disease (CHD) in newborn. There have been various studies showing a number of adverse outcomes, including gestational hypertension, reduced birth weight, altered neonatal pain responses and persistent pulmonary hypertension of the newborn with exposure to anti-depressant medications. There have been very few longitudinal studies showing CHD association with the use of anti-depressant medications. Our objective is to examine the risk for congenital heart disease of the newborn associated with prenatal exposure to antidepressant medication.

Methods

We reviewed charts of mothers who were referred for a fetal echocardiogram between January 1^st^, 2009 and December 31^st^, 2014. We identified mothers who were exposed to antidepressant medications prenatally. Fetal echocardiograms for these patients were reviewed by two fetal cardiologists and each was blinded to the others' findings.

Results

A total of 40 patients were identified with prenatal exposure to SSRI. Seven (18%) out of these 40 were found to have a form of CHD. Two fetuses whose mothers were exposed to fluoxetine during pregnancy had large posteriorly malaligned ventricular septal defect, sub-aortic stenosis and critical coarctation identified on fetal echocardiogram. Exposure to citalopram during pregnancy was found to be associated with a moderate size secundum atrial septal defect on one patient and a moderate size mid muscular ventricular septal defect seen on fetal echocardiogram in another patient. Exposure to venlafaxine during pregnancy showed two small muscular ventricular septal defects on fetal echocardiogram on one patient and ductal constriction with increased ductal velocity on another patient. One of the women on escitalopram had a fetus with a large membranous ventricular septal defect (VSD), secundum atrial septal defect (ASD) and left superior vena cava. None of the women on a combination of drugs had CHD.

Conclusion

There is a risk of congenital heart disease in patients who are prenatally exposed to anti-depressant medications as evident by the specific echocardiographic abnormalities noted in the study.

## Introduction

Maternal depression during pregnancy is a major health care problem, as approximately 10 to 20 percent of women experience depression during their pregnancy [1]. The selective serotonin reuptake inhibitors (SSRIs) and serotonin-norepinephrine reuptake inhibitors (SNRIs) have become the mainstay of pharmacologic treatment for maternal depression during pregnancy [1-4]. It is unclear, however, whether these agents pose a risk to the fetus.

Placental passage of antidepressants has been documented in human population. Fluoxetine and citalopram had the highest ratio of umbilical vein-to-maternal serum concentration, indicating greater transfer from mother to fetus. Sertraline and paroxetine had the lowest ratio [5, 6].

Newborns exposed to maternal SSRI have increased risk of prematurity, low birth weight, persistent pulmonary hypertension of newborn, decreased Apgar score and increased neonatal intensive care/special care nursery admissions. Third trimester exposure to SSRIs increases the risk of respiratory distress syndrome, feeding difficulties, hyperbilirubinemia and neonatal convulsion [7]. Furthermore, there is a higher incidence of preterm birth in women with depression taking SSRI compared to the women with depression not taking SSRI [8].

In recent years, an increasing body of knowledge has been reported challenging the safety of these drugs during pregnancy. The FDA acknowledges most of the SSRIs and SNRIs as being class C. The official report from the American Psychiatric Association and the American College of Obstetrics and Gynecology states that serotonin reuptake inhibitor usage during pregnancy has been associated with miscarriages, premature and/or low birth weight infants and fetal malformations [1]. Several studies have reported that paroxetine (class D) exposure during the first trimester of pregnancy is associated with fetal cardiac abnormalities such as septal defects, right ventricular outflow tract obstruction defects, left ventricular outflow tract obstruction defects and conotruncal abnormalities [1, 2, 4, 9]. But these findings have not been reproduced in larger prospective trials [10-12]. Another report has indicated that antenatal use of SSRIs, in conjunction with benzodiazepine, is linked to an increase in cardiac maldevelopment, as opposed to SSRIs alone [13]. Other studies suggest an increased risk of pulmonary hypertension in newborns, with the use of SSRIs during the third trimester of gestation [14-16]. In our investigation, we focused on detection of echocardiographic abnormalities in fetuses exposed to SSRIs and SNRIs. These findings were then verified with the performance of postnatal echocardiograms.

## Materials and methods

A retrospective review of the institutional medical records at Children’s Hospital of New Orleans, from Jan 1^st^ 2009 to December 31^st^ 2014, identified all pregnant women who underwent fetal echocardiography because of an in-utero exposure to either SSRIs or SNRIs. Women who themselves possessed a known congenital heart defect or a genetic abnormality were excluded. We also excluded women taking benzodiazepines or tricyclic antidepressant class of medications. The following information was obtained: age of the mother, anti-depression medications being used, gestational age at the time of the fetal echocardiogram and the echocardiographic findings. Two experienced fetal cardiologists independently reviewed the echocardiograms, and each was blinded to the other’s findings. The institutional review board of Louisiana State University Health Science Center, New Orleans, LA approved this study.

## Results

**Figure 1:**
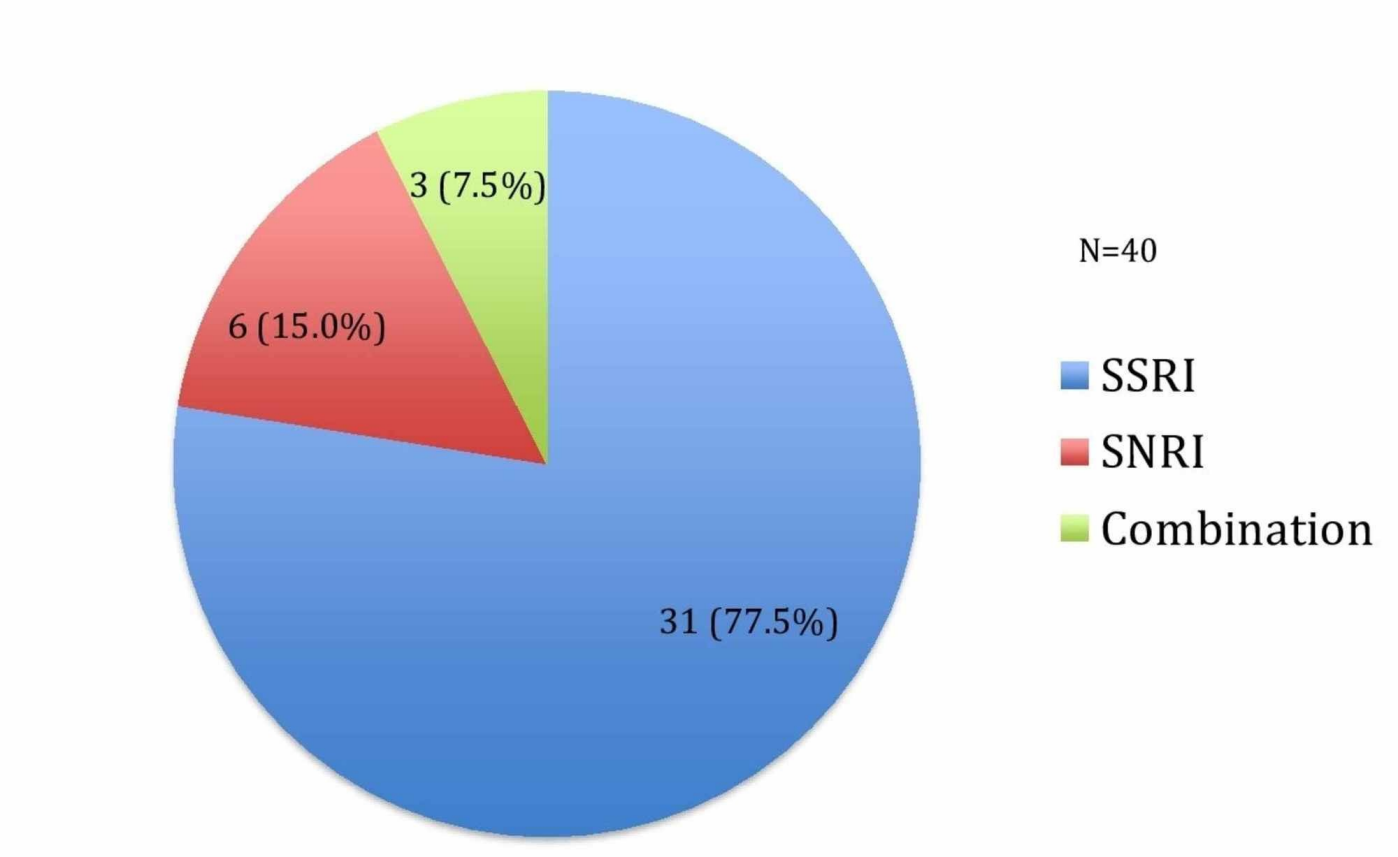
Percentage (%) of mothers on anti-depressant during pregnancy. SSRI: Selective serotonin reuptake inhibitor; SNRI: Serotonin-norepinephrine reuptake inhibitor.

A total of 40 pregnant women were identified, who had undergone a fetal echocardiogram because of in utero exposure of the fetus to SSRIs or SNRIs. Figure 1 shows the percentage of women who were taking each antidepressant. Thirty-one of these women (77.5%) were receiving SSRIs, six (15%) SNRIs and three (7.5%) were on a combination of drugs.

Among the 31 women being treated with SSRIs, five were on citalopram, nine were on escitalopram, nine were on fluoxetine, seven were on sertraline and one was on paroxetine. Among the six women treated with SNRIs, two were on venlafaxine, three were on bupropion and one was on duloxetine. Three women were on a combination of anti-depression medications. The combinations consisted of fluoxetine and bupropion, sertraline and paroxetine, and venlafaxine and bupropion.

Among the 40 women who had fetal echocardiograms, seven (18%) were found to have a fetus with congenital heart disease (CHD). Table 1 outlines the cardiac diagnoses of these fetuses.

**Table 1:** Gestational age at which congenital heart disease diagnosed in utero, type of antidepressant use, echocardiographic findings in the fetuses. VSD: Ventricular septal defect; ASD: Atrial septal defect; L-SVC: Left superior vena cava.

Patient number	Gestational age at echo (weeks)	Anti-depressant medications during pregnancy	Fetal echocardiography findings
1	19.5	Fluoxetine	Large posteriorly malaligned VSD, subaortic stenosis, critical coarctation of aorta
2	19	Fluoxetine	Large posteriorly malaligned VSD, subaortic stenosis, critical coarctation of aorta
3	25	Citalopram	Moderate size mid-muscular VSD
4	27	Venlafaxine	Two mid-muscular VSDs
5	33.5	Venlafaxine	Ductal constriction with doppler velocity of 2.38 m/sec
6	23.5	Citalopram	Moderate size secundum ASD
7	28.3	Escitalopram	Large membranous VSD, secundum ASD and L-SVC

**Figure 2:**
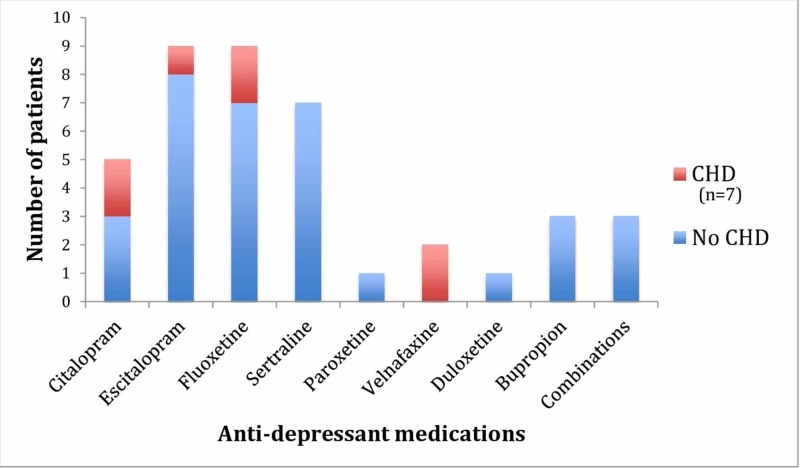
Proportion of patient with CHD and anti-depressant exposure. CHD: Congenital heart disease

Figure 2 shows the incidence of congenital heart disease relative to each medication. Five of the 31 women (16%) on SSRIs and two of the six women (33%) on SNRIs were found to have a fetus with cardiac abnormalities by fetal echocardiogram. Two of the women on fluoxetine, at 19 and 19.5 weeks of gestation respectively, were determined to have the same combination of cardiac abnormalities, namely, a large posteriorly malaligned ventricular septal defect (VSD), sub-aortic stenosis and underdevelopment of the aortic arch. In one of the women on citalopram at 25 weeks, the fetus was found to have a moderate-size mid-muscular VSD, and in another woman on the same drug, at 23.5 weeks, the fetus was found to have a moderately-large secundum atrial septal defect (ASD). In one of the women on venlafaxine at 33.5 weeks, constriction of the ductus arteriosus was detected with a ductal Doppler flow velocity of 2.4 m/sec, and in another woman on the same drug, at 27 weeks, the fetus was found to have two mid-muscular VSDs. One of the women on escitalopram had a fetus with a large membranous VSD, secundum ASD and left superior vena cava. None of the women on a combination of drugs had CHD.

The two fetal echocardiographers reviewed the studies independently and arrived at the same conclusion. In each case, the cardiac abnormalities diagnosed in utero were confirmed following birth, with a postnatal transthoracic echocardiogram.

## Discussion

The use of anti-depressive medications during pregnancy has increased steadily over the years, with a reported prevalence of 8 to 13% in the United States. Herein, we report six-year experience of fetal echocardiographic findings in pregnant women treated with SSRIs, SNRIs or combination antidepressants. We found fetal echocardiographic abnormalities in 18% of the fetuses, whereas the incidence of CHD is 1% in general population. According to data from Centers for Disease Control and Prevention (CDC), occurrence of ASD, VSD and coarctation of aorta is 13, 42 and 4 per 10,000 children, respectively. Two of the fetuses were diagnosed with major congenital heart defects requiring cardiac surgery during the newborn period. Four other fetuses were diagnosed with septal defects - ASDs and VSDs. One fetus was diagnosed with ductal constriction secondary to venlafaxine, an SNRI without any known direct cardiac effects; this fetus was not exposed to non-steroidal anti-inflammatory drugs.

A meta-analysis conducted by Myles et al. did not find any congenital malformation in mothers who were on citalopram during their pregnancy [1]. In our study, citalopram was associated with septal defects in two cases. We also did not find any CHD in case of mother taking paroxetine, although we only had one woman out of 40 taking paroxetine. In several studies, paroxetine has been found to be consistently associated with CHD and its risk reflected when, in 2005, FDA issued a public health advisory on its use in first trimester [1, 4, 17]. In our study, fluoxetine was associated with cardiac malformations, which was also consistent with previous study [1].

Like most other epidemiologic studies, our study also had its own strengths and limitations. Our study was a retrospective review, single center study and had a relatively small sample size. Moreover, the study did not take into consideration of maternal intake of other medications such as anti-seizure medications or oral hypoglycemics, and its effect on overall association with infant with CHD. We also did not review the history of CHD in expectant mothers' other children. Despite these limitations, our study had several strengths such as confirmation of anti-depressant medications through chart reviews rather than telephonic interviews. Our study also confirmed the fetal echocardiographic findings with postnatal findings. Additionally, all of the echocardiographic findings were read by two independent fetal cardiologists.

## Conclusions

There is a possible association of CHD in patients who are prenatally exposed to SSRIs or SNRIs. Until we have prospective long-term safety studies, careful risk-benefit analysis needs to be applied when considering the use of SSRIs or SNRIs in pregnancy.
